# Improving Weak Subgrade Soil Using Different Additives

**DOI:** 10.3390/ma15134462

**Published:** 2022-06-24

**Authors:** M. S. Eisa, M. E. Basiouny, A. Mohamady, M. Mira

**Affiliations:** 1 Civil Engineering Department, Benha Faculty of Engineering, Benha University, Benha 13518, Egypt; mohamed.basiouny@su.edu.eg; 2 El Arish Faculty of Engineering, Sinai University, El-Arish 45511, Egypt; 3 Construction Engineering and Utilities Department, Faculty of Engineering, Zagazig University, Zagazig 44519, Egypt; dr_amohamady@yahoo.com; 4 Benha Faculty of Engineering, Benha University, Benha 13518, Egypt; mahmoudmira2024@gmail.com

**Keywords:** swelling, clayey soil, subgrade strength, granular stabilization, chemical stabilization, additives, consistency tests, CBR test, (C-D) tri-axial shear test

## Abstract

Weak subgrade is the main problem facing most highway projects. Therefore, this study focuses on trying to improve the properties and increase the strength of weak, clayey, swelling soil for use as a subgrade for pavement structural sections. This trial was developed using a mix of granular and chemical stabilization for the soil. Granular stabilization was applied firstly by mixing natural sand at different percentages of 20%, 35%, and 50% of the total weight of clayey, swelling soil samples to find the minimum percentage that could be added to improve it to sandy, clayey soil, which is acceptable as a subgrade according to the Egyptian highway specification code. Secondly, chemical stabilization was applied to enhanced sandy, clayey soil to increase its strength properties. This was performed by adding chemical additives (lime, cement kiln dust (CKD), fiberglass, Addicrete 11, and gypsum) at different ratios of 2%, 4%, and 6% of the total weight of the samples of enhanced sandy, clayey soil. An experimental program was conducted consisting of characteristics and consistency tests, the California bearing ratio (CBR) test, a proctor test, and a consolidated-drained (C-D) tri-axial shear test. The results showed that 50% sand was the minimum percentage that could be mixed with swelling, clayey soil for granular stabilization to be enhanced and become sandy, clayey soil, which is accepted as a subgrade layer according to the Egyptian highway specification code. In addition, using a mix of granular and chemical stabilization increased the compressive strength of this enhanced subgrade by adding 6% lime or cement kiln dust (CKD) of the total sample weight. They enhanced the strength of the soil and reduced its plasticity. Adding 6% fiberglass and polymers could slightly enhance the desired properties; however, it is not recommended to use them due to their slight effect and economic cost. In addition, it is not recommended to use gypsum at more than 4% due to its negative effect on CBR.

## 1. Introduction and Background

The national road network is the most important project undertaken recently by the Egyptian government. It is considered the most important main access that is dependent on the occurrence of economic development because of its important role in facilitating the movement of trade and linking the industrial, agricultural, tourism, airport, seaport, and housing communities together. The government needs to speed up the completion of these projects in a very short time to achieve the desired goal. Due to this rapid completion, some roads are required to be constructed in places that are not well-suited as subgrade for pavement sections. For example, some types of soils are very soft clay or swelling soil. As a result, some defects in the wearing surfaces of the pavement sections of these roads have recently been observed, such as those seen recently in the Ismailia–Port Said highway. Therefore, many studies have been carried out to try and evaluate solutions to this problem in new ways. This study is one of them.

Based on the previous studies found that many researchers have carried out in this field, most have used sand only as granular stabilization. Kollars and Athanasopoulou [1] found that up to 60% sand by weight of the soil could be added to enhance swelling soil, while Nair and Salini [2] showed that 50% could be added to enhance swelling, clayey soil to be acceptable as a subgrade. Sometimes, this has led to having a larger amount of sand, depending on the type of soil, to improve the properties of weak, swelling soil. However, it may be not an economical solution for most cases, and it may not be a suitable solution to give the desired characteristics. Therefore, a mix of granular and chemical stabilization may be the most preferable solution to gain the desired properties. On the contrary, with an economical view and demanding greater strength resistance, especially for large highway projects, it is recommended to mix different additives to enhance clayey soil with sand. Kollars and Athanasopoulou [1] recommended this in their study. Therefore, in this study, different additives (lime, cement kiln dust (CKD), fiberglass, Addicrete 11, and gypsum) with different ratios are added to swelling, clayey soil enhanced with sand. Both Daipuria and Trivedi [3] and Ramteke et al. [4] found that adding 20% to 40% sand mixed with 2% cement increased the soil resistance strength. Other researchers have added CKD to swelling soil, as Keerthi et al. [5] found that using up to 50% CKD added to swelling soil could increase its strength [5]. Afaf et al. [6] proved that 16% CKD improved the stabilization of expansive soil in the Sohag region, Egypt, while Mosa et al. [7] exhibited that adding 20% CKD with curing for 14 days improved the properties of poor subgrade soil.

Lime has also been used in many studies to improve the characteristics of swelling, clayey soil. Both Hesham et al. [8] and Afaf et al. [6] showed that mixing 5–6% lime of the total soil weight gave satisfactory results for swelling soil in the subgrade of the Upper Egypt–Red Sea Road. In addition, Chen [9] found that the amount of lime required to stabilize expansive soils ranged from 2–8% by weight. Nair and Salini [2] showed in their study that adding 1% lime to problematic soil mixed with 50% sand gave good properties of the soil.

Gypsum is used in this study to try to increase soil strength. Murthy et al. [10] added 25% gypsum to the total weight of silty clay. They found improvement in the soil properties.

Fiberglass and Addicrete 11 are two kinds of polymers that are investigated in this study for the stabilization of swelling soil. El-Kasaby [11] showed that mixing 0.6% of the total weight of fiberglass mesh with swelling soil increased the cohesion, internal friction, and compressive strength of the soft clay. Hashem et al. [12] found that adding 6% fiberglass or Addicrete 11 to native soil improved the soil properties.

Based on the literature review, this study is carried out through two approaches. The first one is enhancing swelling, clayey soil using granular stabilization by mixing different ratios of sand (20%, 35%, and 50% of the sample total weight) to create an acceptable subgrade. The second approach is chemical stabilization to increase the strength of the enhanced subgrade soil by adding other additives (lime, CKD, fiberglass, Addicrete 11, and gypsum) at different percentages (2%, 4%, and 6%) of the total sample weight to enhanced clayey soil with sand to obtain the optimum percentages that can be added to improve and increase subgrade resistance to satisfy the desired characteristics. The basis for selecting the dosages of various modifiers is based on previous studies [2,3,4], which have added higher dosages of additives to native soil directly (chemical stabilization only), while in this study, the additives are added in small amounts to enhanced sandy, clayey soil to increase its strength, in addition to having an advantage from an economic viewpoint.

## 2. Tested Materials and Experimental Design

### 2.1. Tested Materials

#### 2.1.1. Natural Soil

Very soft, swelling clay was the natural soil used in this research as a subgrade. It was obtained from road construction near the El-Kasasin village in the Ismailia Governorate, Egypt. It was brought from the excavation from about 2 m deep under the ground’s surface (the foundation level, according to a geotechnical report). It was collected from different locations on the site. Table 1 shows the properties of the natural soil.

#### 2.1.2. Natural Sand

Natural sand was used to enhance the undesired properties of the natural soil (very soft clay) by mixing the soil with different percentages of natural sand. The natural sand was mixed at different percentages (20%, 35%, and 50%) of the total sample weight. Table 1 shows the properties of the natural sand and the properties of the mixes of natural soil enhanced with different percentages of natural sand.

#### 2.1.3. Lime

Lime was one of the additives used in this study that is known commercially as hydrated lime (CaOH_2_) and was produced by the Tura Company (Meghalaya, India). The analysis supplied by the manufacturer is indicated in Table 2.

#### 2.1.4. Cement Kiln Dust (CKD)

Another additive used in this study was ordinary cement kiln dust produced and collected by the Tura factory (Meghalaya, India). The chemical analysis of CKD is given in Table 3. The CKD had a density of 3.08 g/cm^3^, a bulk density of 1.17 g/cm^3^, and a porosity of 0.62.

#### 2.1.5. Fiberglass

Fiberglass was one of the additives used in this study in the form of pure polypropylene short-cut fibers produced with chemicals by the modern building company CMB, Cairo, Egypt. The production data shows that it had a cut length of 15 mm and a density of 0.91 gm/cm^3^.

#### 2.1.6. Addicrete 11

The polymer additive in this study was a brown chemical powder based on polymerized resins produced by the CMB Company, Cairo, Egypt. Its tradename is Addicrete 11. The datasheet from the production factory showed that it had a density of 0.64 ± 0.01 kg/lit. The material is added to cement bricks to obtain a rapid dry rate and to increase the resistance to fracturing.

#### 2.1.7. Gypsum

The last additive used in this study was gypsum. It is a naturally occurring mineral that is made of calcium sulfate and water (CaSO_4_ + 2H_2_O) and is sometimes called hydrous calcium sulfate. Gypsum has 23% calcium and 18% sulphur, its solubility is 150 times that of limestone, and its specific gravity is 2.3. It is mined and made into many products, such as drywall, that are used in construction, agriculture, and industry. It is also a byproduct of any industrial process. It was obtained from the Elblaah Company, Cairo, Egypt.

### 2.2. Experimental Design

**Figure 1:**
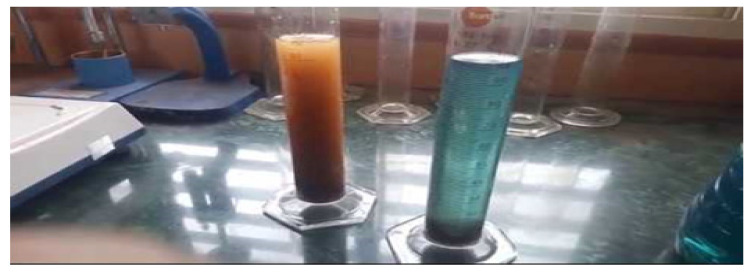
Free swelling test apparatus.

**Figure 2:**
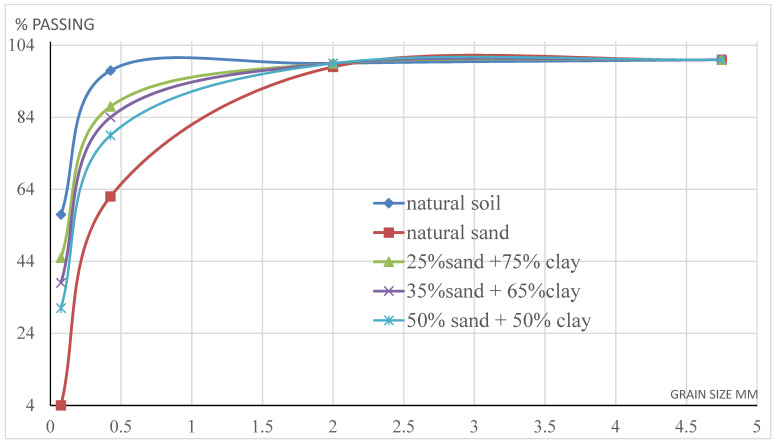
The grading curves of natural soil, natural sand, and the treated soils with different percentages of sand.

**Figure 3:**
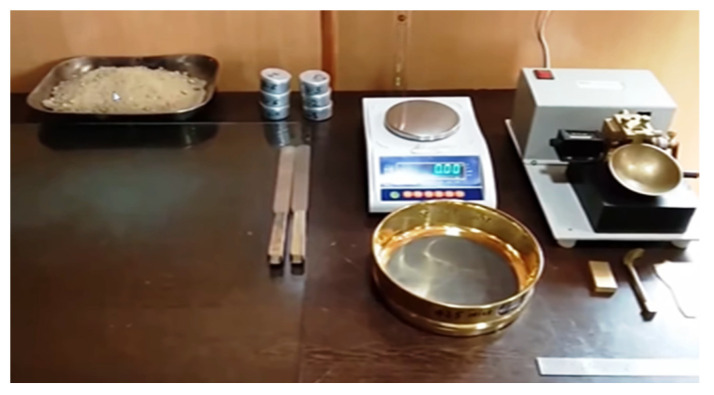
Determination of LL and PL with a Casagrande apparatus.

**Figure 4:**
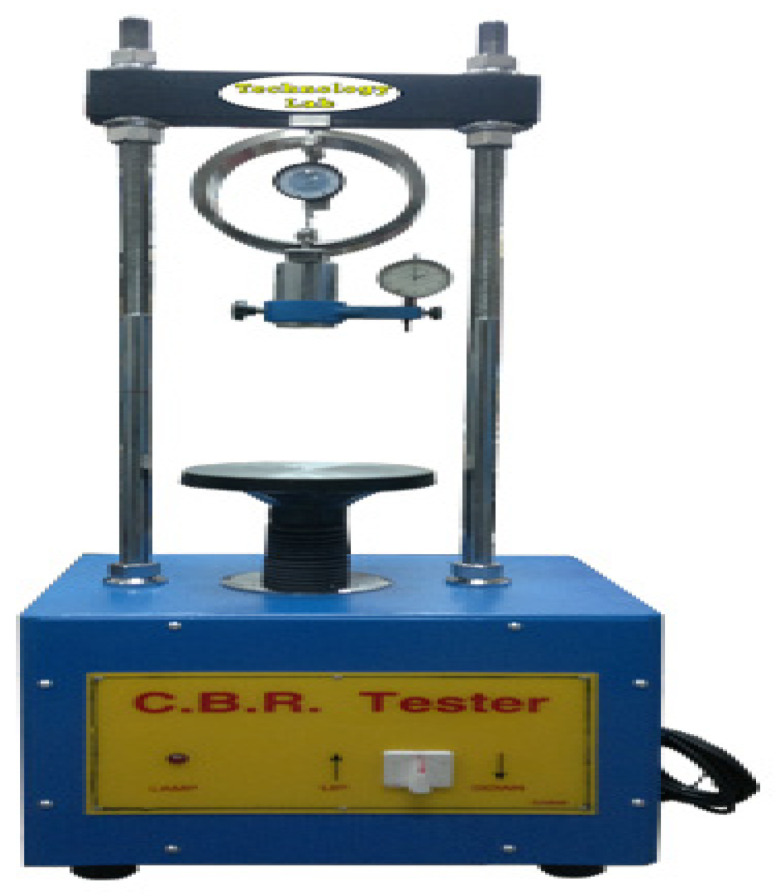
Determination of CBR with a CBR apparatus.

**Figure 5:**
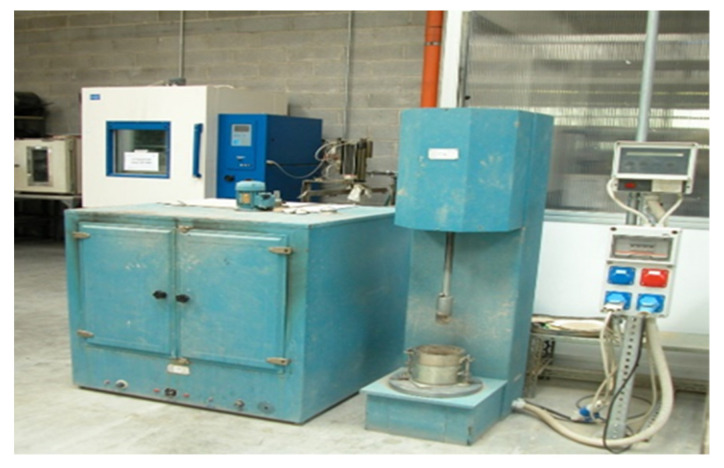
Determination of OMC and MDD with a proctor test.

**Figure 6:**
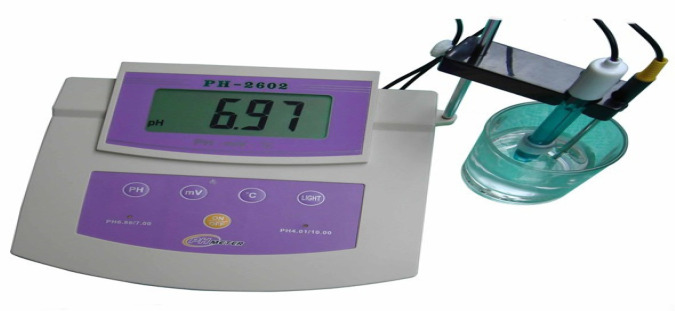
Determination of pH of samples with pH meter.

The goal of the study was to evaluate using a mix of granular stabilization and chemical stabilization to improve weak subgrade clayey, swelling soil properties. Granular stabilization was used firstly in this study by mixing natural soil with different percentages of natural sand, and finally, chemical stabilization was carried out by adding different percentages of additives (lime, CKD, gypsum, fiberglass, and Addicrete 11) to natural soil enhanced with sand to increase the improving properties of the subgrade. To investigate the goals of this study, an experimental program was designed and is described in the following steps:

1. The natural soil samples were air-dried and pulverized to pass through a no. 4 sieve (4.75 mm) and then placed in an oven and left for 24 h at a temperature of 110 °C to control and check the humidity of the sample. Then, a sample (S0) was taken. The basic properties of the sample were determined using a group of tests, including:
  - A free swelling test. Figure 1 shows the determination of the free swelling ratio using a free swelling test apparatus.
  - Grain size distribution according to AASHTO T-27 [13] and a hydrometer analysis. The physical properties of the soil were studied, and the soil was classified as A-7-6 according to the AASHTO classification system, while according to the unified classification system, it was classified as CL. Figure 2 shows the grading curves of natural soil, natural sand, and the treated soils with different percentages of sand.
  - The liquid limit (LL) and plastic limit (PL) using the Casagrande method. Figure 3 shows the determination steps for the LL and PL using a Casagrande apparatus (indiaMart manufacturer, Uttar Pradesh, India).
  - A CBR test. Figure 4 shows the determination of CBR using a CBR apparatus (indiaMart manufacturer, Uttar Pradesh, India).
  - A proctor test to determine the optimum moisture content (OMC) and maximum dry density (MDD) of the soil samples. Figure 5 shows the determination of OMC and MDD using a proctor test.
  - Determining the pH values of the soil samples. Figure 6 shows the determination of the pH values of the soil samples using a pH meter according to AASHTO T 289-91 (2018).

All the tests were carried out according to the Egyptian specification code for soil mechanics [14].

**Figure 7:**
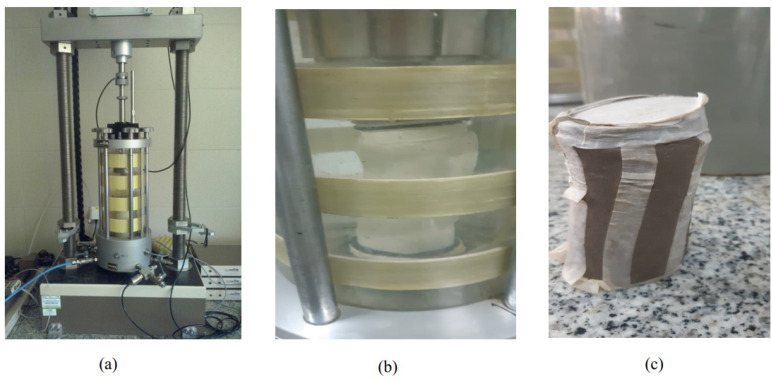
Determination steps for shear parameters (C and Φ) using a tri-axial apparatus. (**a**) Tria-axial test apparatus; (**b**) Soil sample in tri-axial cell; (**c**) Soil sample at failure.

2. Granular stabilization was carried out to enhance the natural, clayey soil by making samples containing mixes of natural, clayey soil and different percentages of natural sand of 20%, 35%, and 50% of the total weight of the sample to make samples S1, S2, and S3, respectively, as shown in Table 1.
3. The group of tests mentioned above was repeated for samples S1, S2, and S3 to obtain the minimum percentage value of sand that could be mixed with natural soil to enhance and achieve the desired and acceptable properties for use as a subgrade, according to the Egyptian highway specification code [15].
4. Chemical stabilization was carried out based on the previous step by making samples containing natural, clayey soil enhanced with the minimum percentage value of sand gained from the previous step and adding additives (CKD, lime, fiberglass, Addicrete 11, and gypsum) at different percentages of 2%, 4%, and 6% of the total weight of the samples to form samples S4–S15, as shown in Table 1.
5. The group of tests mentioned above was repeated and carried out for samples S4–S15 to show the effects of the additives on the properties of enhanced, clayey soil with sand to improve and increase its strength and durability.
6. From step 5, we could obtain the optimum percentages that could be added to enhance natural, clayey soil to improve its strength. Hence, we formed samples at these percentages and applied the consolidated-drained (C-D) tri-axial shear test for these samples to show the effects of the additives on internal fraction and cohesion for particles of natural, clayey soil enhanced with sand for improving its shear strength resistance. Figure 7 shows the determination of the shear parameters (C and Φ) with a tri-axial apparatus.

## 3. Results and Discussion

This section presents the results of the study program tests. An analysis of these results is presented in the following subsections.

### 3.1. Evaluation of Grain Size Distribution

A grain size analysis was carried out for the soil according to AASHTO T-27 [11] and a hydrometer analysis. The natural, clayey soil was classified as A-7-6, and the natural sand used was classified as A-3.

Natural soil mixed with 20% and 35% natural sand to form samples S1 and S2 was classified as A-6, which meant that it was still poor for use as a subgrade, according to the Egyptian highway specification code [15]. The natural soil mixed with natural sand at 50% of the total weight of the sample was classified as A-2-4. Figure 2 shows the grading curves of natural soil, natural sand, and the treated soils with different percentages of sand.

The samples from S4–S18 contained 50% natural soil and 50% natural sand when mixed with different percentages of additives. They were classified as A-2-4 for all the samples.

### 3.2. Effect of Granular Stabilization on Soil Properties

Granular stabilization was carried out by mixing natural sand at different percentages (20%, 35%, and 50%) of the total weight of the samples with natural, clayey soil. It enhanced the properties of natural, clayey soil as shown below.

#### 3.2.1. Effect of Granular Stabilization on Free Swelling Ratio

**Figure 8:**
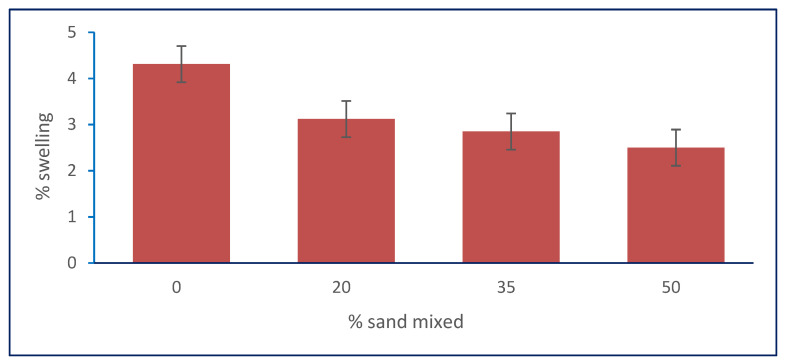
Effect of sand on swelling percentage.

In Table 1, the results show that the free swelling ratio decreased with increasing percentages of sand (20%, 35%, and 50%) mixed with natural, clayey soil. Figure 8 shows the relation between the free swelling ratio and the sand percentages. It shows that the free swelling ratio decreased from 4.31% to its optimum value of 2.5% when the soil was mixed with 50% sand in sample S3. This behavior may be due to a reduction in the percentage of clay in the sample, as well as because fine particles of clay may have filled the voids between particles of sand, leading to a reduction in swelling.

#### 3.2.2. Effect of Granular Stabilization on LL

**Figure 9:**
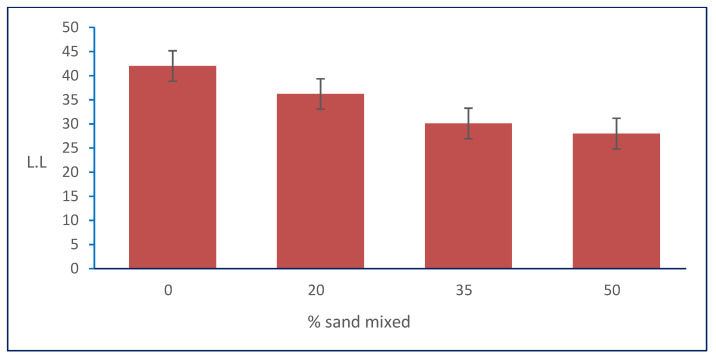
Effect of sand on LL.

In Table 1, the results show that the liquid limit of the natural soil decreased with the increasing percentages of natural sand mixed with it. The LL decreased from 42 to its minimum value of 28 at the percentage of 50% of the total weight of the sample of natural sand added to natural, clayey soil, as shown in sample S3. Figure 9 shows the relation between the liquid limit and sand percentages.

#### 3.2.3. Effect of Granular Stabilization on PL

**Figure 10:**
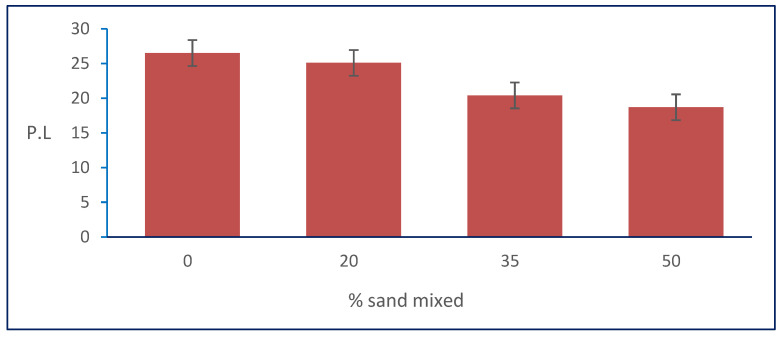
Effect of sand on PL.

The results in Table 1 show that the plastic limit of the natural soil was decreased by increasing the percentages of natural sand mixed with it. Figure 10 shows the relation between the plastic limit and sand percentages. It shows that the PL decreased from 26.5 to its minimum value of 18.7 at the percentage of 50% of the total weight of natural sand mixed with natural, clayey soil for sample S3.

#### 3.2.4. Effect of Granular Stabilization on PI

**Figure 11:**
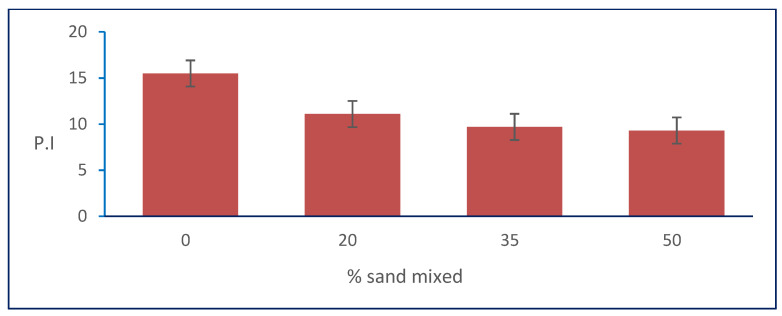
Effect of sand on PI.

In Table 1, the results show that the plastic index of the natural soil was 15.5 and decreased with increasing percentages of natural sand mixed with it. Figure 11 shows the relation between the plasticity index and sand percentages. It shows that the PI decreased from 15.5 to its minimum value of 9.3 at the percentage of 50% of the total sample weight of natural sand mixed with natural, clayey soil for sample S3.

#### 3.2.5. Effect of Granular Stabilization on CBR

**Figure 12:**
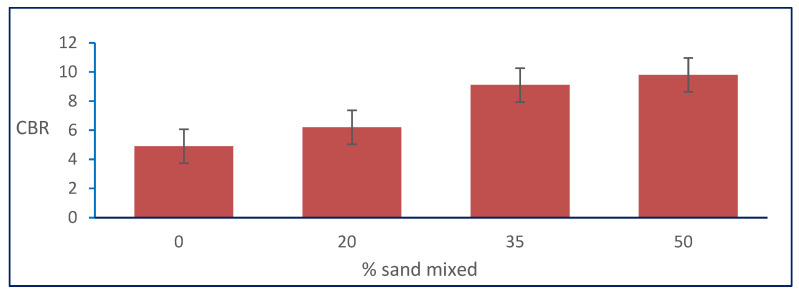
Effect of sand on CBR.

From Table 1, the results show that the CBR of the natural soil was 4.9, and it increased with increasing percentages of natural sand mixed with it. The CBR increased from 4.9 to its maximum value of 9.8 at the percentage of 50% of the total weight of natural sand mixed with natural, clayey soil for sample S3. Figure 12 shows the relation between the California bearing ratio (CBR) and sand percentages.

#### 3.2.6. Effect of Granular Stabilization on OMC

**Figure 13:**
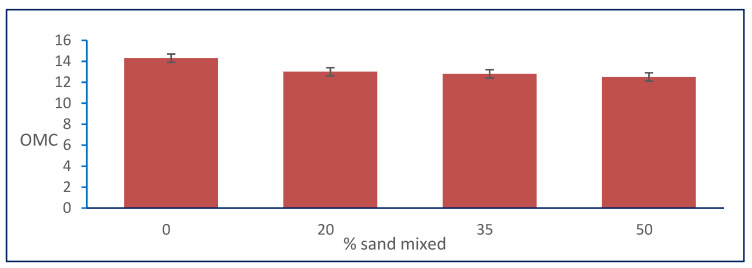
Effect of sand on optimum moisture content.

In Table 1, the results show that the OMC of the natural soil was 14.3, and it decreased with increasing percentages of natural sand mixed with it. The OMC decreased from 14.3 to its minimum value of 12.5 at the percentage of 50% of the total sample weight of natural sand mixed with natural, clayey soil for sample S3. Figure 13 shows the relation between the optimum moisture content (OMC) and sand percentages.

#### 3.2.7. Effect of Granular Stabilization on MDD

**Figure 14:**
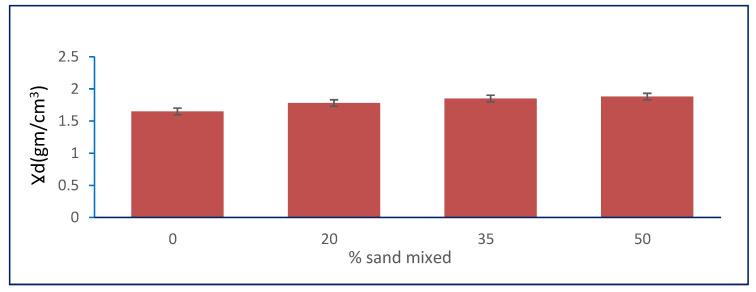
Effect of sand on maximum dry density.

The Table 1 results show that the MDD of the natural soil was 1.65, and it increased by increasing the percentages of natural sand mixed with it. The MDD increased from 1.65 to its maximum value of 1.86 at the percentage of 50% of the total sample weight of natural sand added only to natural, clayey soil in sample S3. Figure 14 shows the relation between the maximum dry density and sand percentages.

### 3.3. Effect of Chemical and Granular Stabilization on Soil Properties

Based on the results obtained in this study mentioned above, it was shown that sample S3, a sandy, clayey soil that consisted of 50% sand and 50% clay, had the most enhanced properties of the soil. Therefore, the minimum percentage of sand that could be mixed with the natural, clayey soil was 50%. It could enhance the soil properties to be acceptable as a subgrade in pavement structural sections, according to the Egyptian highway specification code [15], as shown in sample S3. Hence, chemical stabilization was carried out for sample S3 by adding additives (CKD, lime, fiberglass, Addicrete 11, and gypsum) at different percentages of 2%, 4%, and 6% of the total weight of the sample to form samples S4–S15. The following subsections show the effects of combinations of granular and chemical stabilization on soil properties.

#### 3.3.1. Effect of Chemical and Granular Stabilization on Free Swelling Ratio

**Figure 15:**
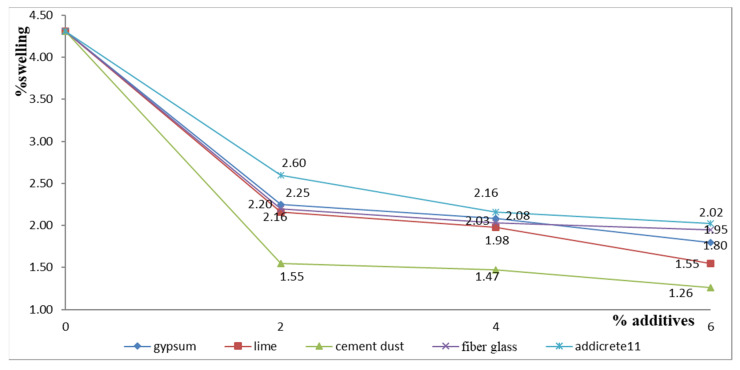
Effects of additives on swelling percentage.

In Table 1 and Figure 15, the results show that the swelling ratio decreased and reached the optimum values of 1.55 and 1.26 when 6% lime and CKD were added to enhanced sandy, clayey samples for samples S6 and S9, respectively, which contained 47% clay, 47% sand, and 6% lime or CKD. Adding fiberglass, gypsum, and Addicrete 11 reduced the swelling ratio, but they had negligible effects on the samples, as they slightly decreased the values to 1.95%, 2.02%, and 1.8%, respectively, when added at 6%.

#### 3.3.2. Effect of Chemical and Granular Stabilization on LL

**Figure 16:**
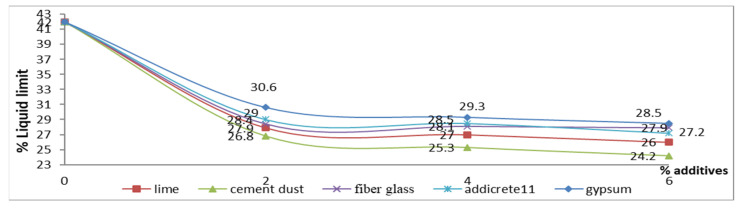
Effect of additives on the liquid limit.

In Table 1 and Figure 16, the results show that the LL decreased with increasing percentages of content for all the additives. However, the highest decreases were remarked for adding CKD and lime, respectively. The LL decreased from 42 to 26 and 24.2 in the cases of adding 6% lime and CKD, respectively, as shown in samples S6 and S9, while slight decreases were remarked of 28, 27.9, and 27.2 for the cases of adding 6% gypsum, fiberglass, and Addicrete 11, respectively. Figure 16 shows the relationship between the additive percentages added to enhanced sandy, clayey soil and the LL. It displays nonlinear curves, showing that the LL decreased with increasing percentages of content for all the types of additives.

#### 3.3.3. Effect of Chemical and Granular Stabilization on PL

**Figure 17:**
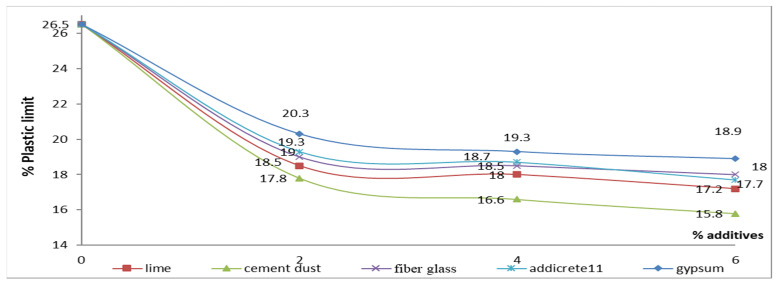
Effect of additives on the plastic limit.

In Table 1 and Figure 17, the results show that the PL decreased with increasing percentages of all the additives. The highest decreases were remarked for adding CKD and lime, respectively. The PL decreased from 26.5 to 17.2 and 15.8 in the cases of adding 6% lime and CKD, respectively, as shown in samples S6 and S9. In addition, decreases were remarked of 18.9, 18, and 17.7 for the cases of adding 6% gypsum, fiberglass, and Addicrete 11, respectively. Figure 17 shows the relationship between the additive percentages added to enhanced sandy, clayey soil and the PL through nonlinear curves that show that the PL decreased with the increasing percentages of content for all the types of additives.

#### 3.3.4. Effect of Chemical and Granular Stabilization on PI

**Figure 18:**
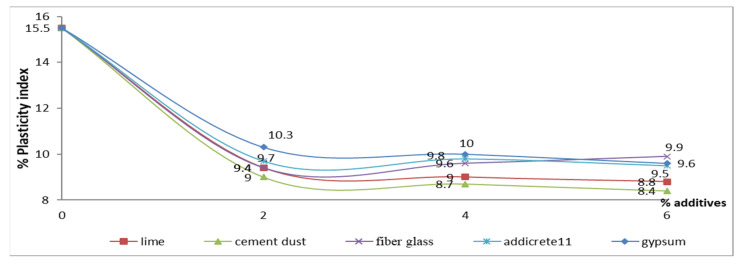
Effect of additives on plasticity index.

In Table 1 and Figure 18, the results show that the PI decreased with increasing percentages of all the additives. The highest decreases were remarked for adding CKD and lime, respectively. The PI decreased from 15.5 to 8.8 and 8.4 in the cases of adding 6% lime and CKD, respectively. However, no significant differences were observed at 9.6, 9.5, and 9.9 in the cases of adding 6% gypsum, Addicrete 11, and fiberglass, respectively. In the case of fiberglass, an increase was observed when the 2% and 4% samples were compared. Figure 18 shows the relationship between the additive percentages added to enhanced sandy, clayey soil and the PI. It shows nonlinear curves that show that the PI decreased with increasing additive percentage content.

#### 3.3.5. Effect of Chemical and Granular Stabilization on CBR

**Figure 19:**
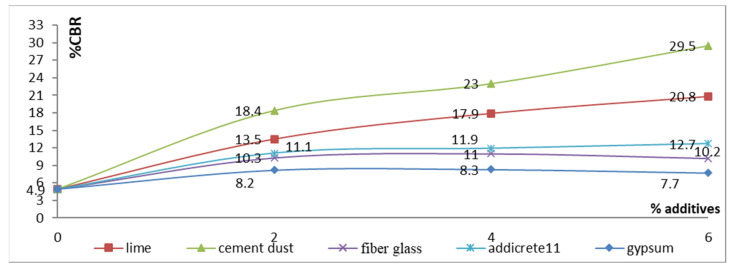
Effect of additives on CBR.

In Table 1 and Figure 19, the results show that the CBR increased with increasing percentages of content for all the additives. The highest decreases were remarked for adding CKD and lime, respectively. The CBR increased from 4.9 to 20.8 and 29.5 in the cases of adding 6% lime and CKD, respectively. This behavior was suggested to be due to the chemical bond produced by the reactions of CKD or lime with components of the soil. Increasing the dose increased the bond, leading to an increase in strength and, consequently, an increase in the CBR. In addition, very slight increases were noticed of 10.2 and 12.7 for the cases of adding 6% fiberglass and Addicrete 11, respectively. The worst results were shown in the case of adding 6% gypsum. As a result of gypsum dissolution and its interaction when immersed in water, a CBR was shown of 7.7 with 6% gypsum. Figure 19 shows the relationship between the additive percentages added to enhanced sandy, clayey soil and the CBR. It is represented as nonlinear curves, showing that the CBR increased with increasing percentages of content for all the additives.

#### 3.3.6. Effect of Chemical and Granular Stabilization on MDD

**Figure 20:**
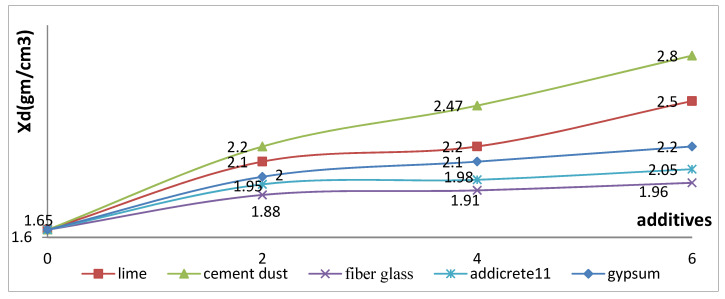
Effect of additives on maximum dry density.

In Table 1 and Figure 20, the results show that, with an increase in the percentages of the additives, the MDD of the soil increased for all the additives. Higher increases were remarked for adding lime and CKD, respectively. The MDD was increased from 1.65 to 2.5 and 2.8 in the cases of adding 6% lime and CKD, respectively. In addition, slight increases were remarked of 2.2, 2.05, and 1.96 for the cases of adding 6% gypsum, Addicrete 11, and fiberglass, respectively. Figure 20 shows the relationship between the additive percentages added to enhanced sandy, clayey soil and the MDD. It demonstrates with nonlinear curves that the MDD increased with increasing percentages of content for all the additives.

#### 3.3.7. Effect of Chemical and Granular Stabilization on OMC

**Figure 21:**
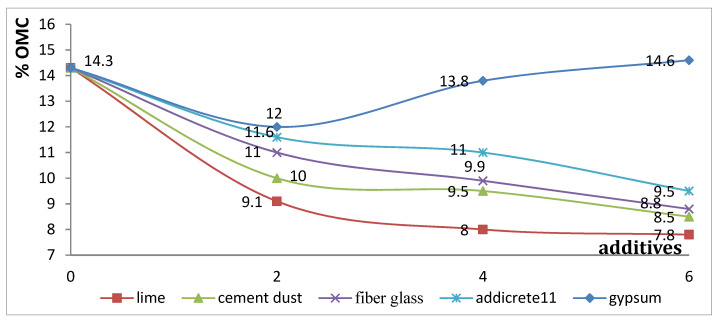
Effect of additives on optimum moisture content.

In Table 1 and Figure 21, the results show that, with an increase in the percentages of the additives, the OMC of the soil decreased for all the additives, except in the case of adding gypsum. Higher decreases were remarked for adding lime and CKD, respectively. The OMC decreased from 14.3% to 7.8% and 8.5% in the cases of adding 6% lime and CKD, respectively. However, it was noticed that, in the case of adding 6% fiberglass, it was decreased to 8.8. The reason for such a behavior was that, due to the replacement of fiberglass particles with soil particles, the attraction for water molecules decreased and, hence, the OMC decreased. In the case of adding gypsum, the OMC increased slightly with increasing added percentages due to the ability of gypsum to absorb water initially and react with it. Figure 21 shows the relationship between the additive percentages added to enhanced sandy, clayey soil and the OMC. It is represented as nonlinear curves, showing the OMC decreased with increasing percentages of content for all the additives, except for gypsum, when the OMC decreased first at percentages of 2% and 4% and then started to increase at 6%.

#### 3.3.8. Effect of Chemical and Granular Stabilization on pH value

**Figure 22:**
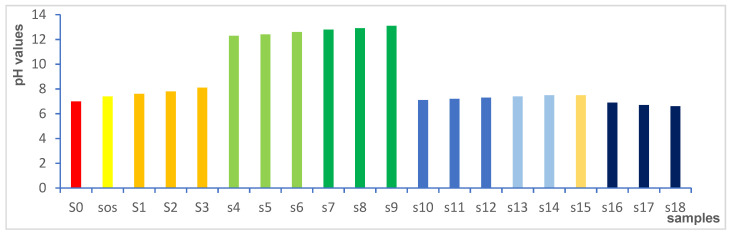
Effect of chemical and granular stabilization on soil sample pH values.

In Table 1 and Figure 22, the results show that the pH value of the natural soil was 7 within a neutral phase, while the pH for natural sand was 7.4. Granular stabilization through mixing different percentages of sand with natural soil slightly increased the pH value of the natural soil from 7.4 to 8.1 at 50% sand. Chemical and granular stabilization achieved the maximum increments of pH value of 12.6 and 13.1 within the alkaline phase at 6% added lime and CKD, respectively. In addition, slight increases were remarked of 7.3 and 7.5 for the cases of adding 6% fiberglass and Addicrete 11, respectively. However, decrease in pH was noticed with increasing percentages of added gypsum. It decreased to 6.6 to enter the acidic phase at 6% added gypsum.

### 3.4. Evaluation of Shear Strength (Consolidated-Drained (C-D) Tri-Axial Test)

Based on the results obtained in this study mentioned above, a consolidated-drained (C-D) tri-axial test was carried out on natural soil (S0), a sample of clayey soil enhanced with sand (S3), and samples of enhanced sandy, clayey soil mixed with 6% lime and CKD (respectively, S6 and S9) to evaluate and compare the effect of granular stabilization only and the mix of chemical and granular stabilization on the shear strength of the subgrade soil. Table 3 shows this comparison by showing the effects of lime and cement kiln dust on the shear strength parameters (C and Φ).

#### 3.4.1. Evaluation of Cohesion

**Figure 23:**
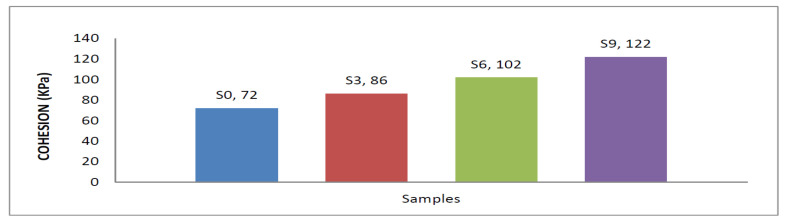
Effects of lime and cement dust on cohesion strength of swelling soil.

In Table 3 and Figure 23, the results show that the cohesion of natural, clayey soil (S0) was 72 kPa. It increased to 86 kPa after granular stabilization when mixed with sand at 50% of the total weight of sample S3. Adding 6% of the total weight of lime and CKD to enhanced sandy, clayey soil samples S6 and S9 improved the cohesion. It was increased up to 102.78 kPa and 122.96 kPa, respectively. This was due to a chemical reaction effect between particles of lime and CKD the enhanced natural soil at the OMC. It generated bonds among the particles, induced the flocculation of soil particles, and filled the voids.

#### 3.4.2. Evaluation of Internal Friction Angle

**Figure 24:**
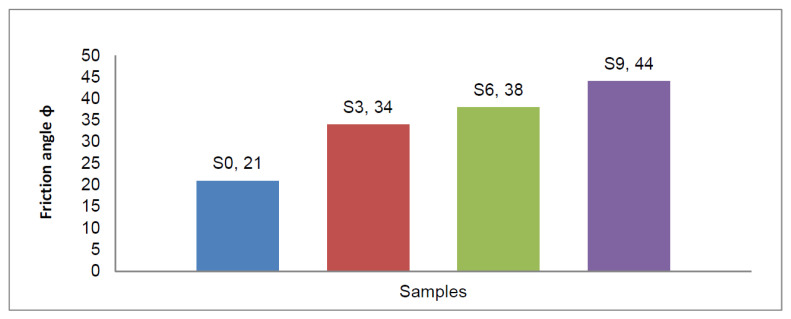
Effects of lime and cement dust on friction angle of swelling soil.

In Table 3 and Figure 24, the results show that the friction angle of natural, clayey soil (S0) was 21°, and it increased to 34° when enhanced by adding sand at 50% of total weight (S3), increasing to 38° and 44° when 6% lime and CKD were added to enhanced sandy, clayey soil samples S6 and S9.

#### 3.4.3. Evaluation of Shear Strength

**Figure 25:**
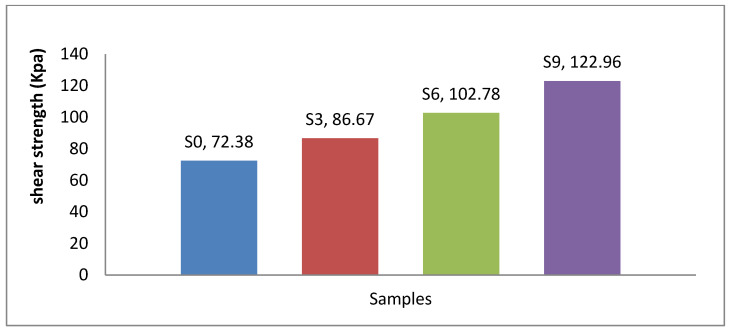
Effects of lime and cement dust on shear strength of swelling soil.

From Table 3 and based on the results of the cohesion and friction angles, the shear strength was obtained according to Coulomb’s law and is shown in Figure 25, which shows that the shear strength increased from 72.38 kPa to 86.67 kPa for the sample enhanced with sand at 50% of the total weight of the sample. The shear strength increased to 102.78 kPa and 122.96 kPa when adding 6% lime and CKD, respectively, to enhanced sandy, clayey soil samples S6 and S9.

## 4. Conclusions and Recommendations

### 4.1. Conclusions

Based on the methodology and the analysis of the results of this study, the following conclusions were drawn:

1. By using granular stabilization, the minimum percentage of sand that could be mixed with natural, clayey, swelling soil was 50% of the total sample weight. It enhanced the clayey soil properties and gradation to be acceptable as a subgrade of pavement, according to the Egyptian highway specification code.
2. Chemical stabilization by adding different additives, such as lime, CKD, fiberglass, gypsum, and Addicrete 11, at different percentages of the total weight could be combined with granular stabilization to increase the improvement of swelling, clayey soil properties, such as the swelling effect, liquid limit, and plastic limit.
3. The best improvement of the clayey soil properties was obtained using a combination of granular and chemical stabilization achieved by mixing 47% natural, clayey soil, 47% natural sand, and 6% lime or CKD. Using other additives, such as fiberglass, gypsum, and Addicrete 11, had a slight positive effect on improving the soil properties.
4. The plasticity index of swelling, clayey soil was enhanced by mixing with 50% natural sand. It was decreased by almost 40% from its original value. Adding 6% lime and CKD increased this improvement, while other additives decreased it. Fiberglass had a lower effect due to no interactions between particles of the sample, while Addicrete 11 and gypsum had almost the same effect.
5. Enhanced swelling, clayey soil mixed with 50% natural sand enhanced the CBR. It was increased by almost 100%from its original value. Adding 6% lime and CKD increased this improvement, increasing the CBR by 324.4% and 502% from its original values, respectively. Additives such as fiberglass and Addicrete 11 had a slight effect, but adding gypsum had a negative effect when increased to adding values more than 4% because it interacted with the water content in the soaked CBR test.
6. The maximum dry density of enhanced swelling, clayey soil mixed with 50% natural sand was increased from 1.65 gm/cm^3^ to 1.86 gm/cm^3^. In addition, it increased with the increasing of all the additive percentages up to optimum values of 2.8 g/cm^3^ and 2.5 gm/cm^3^ when adding 6% CKD and lime, respectively.
7. The optimum moisture content of enhanced swelling, clayey soil mixed with 50% natural sand decreased by 12.5% from its original value. In addition, it decreased with the increase of all the additive percentages, except in the case of gypsum. It began to increase due to the chemical interaction between gypsum and its absorption ability for water content.
8. Enhanced swelling, clayey soil mixed with 47% natural sand and 6% CKD had a larger shear strength resistance than that mixed with 6% lime.
9. The optimum desired properties of natural, clayey soil for use as a subgrade in pavement sections were gained when mixing 47% natural, clayey soil, 47% natural sand, and 6% lime or CKD. However, using 6% CKD gave more improvement than using 6% lime.

### 4.2. Recommendations

Based on the previous conclusions, the following recommendations can be drawn:

1. An evaluation of adding higher percentages of these additives to show their effects on the performance of enhanced swelling, clayey soil with 50% natural sand;
2. An investigation of the performance of using a combination of 6% CKD and lime added to enhanced sandy, clayey soil as a trial to obtain the most suitable enhanced soil properties for use as a subgrade;
3. An economical evaluation using other additives that can be added to enhance swelling, clayey soil mixed with 50% natural sand to obtain the most useful additives from an economical viewpoint;
4. A study on the effect of using these additives with enhanced sandy, clayey soil on the performance of structural pavement sections.

## Figures and Tables

**Table 1:** Properties of samples tested.

		Samples																			
	Symbol	S0	sos	S1	S2	S3	S4	S5	S6	S7	S8	S9	S10	S11	S12	S13	S14	S15	S16	S17	S18
**% percentages**	**natural soil**	100		80	65	50	49	48	47	49	48	47	49	48	47	49	48	47	49	48	47
	**sand**		100	20	35	50	49	48	47	49	48	47	49	48	47	49	48	47	49	48	47
	**lime**						2	4	6												
	**CKD**									2	4	6									
	**fiberglass**												2	4	6						
	**Addicrete 11**															2	4	6			
	**gypsum**																		2	4	6
**properties**	LL	42	0	36.2	30.1	28	27.9	27	26	26.8	25.3	24.2	28.4	28.1	27.9	29	28.5	27.2	30.6	29.3	28.5
	PL	26.5	0	25.1	20.4	18.7	18.5	18	17.2	17.8	16.6	15.8	19	18.5	18	19.3	18.7	17.7	20.3	19.3	18.9
	PI	15.5	0	11.1	9.7	9.3	9.4	9	8.8	9	8.7	8.4	9.4	9.6	9.9	9.7	9.8	9.5	10.3	10	9.6
	CBR	4.9	7.6	6.2	9.1	9.8	13.5	17.9	20.8	18.4	23	29.5	10.3	11	10.2	11.1	11.9	12.7	8.2	8.3	7.7
	Ɣd (gm/cm^3^)	1.65	1.86	1.78	1.85	1.88	2.1	2.2	2.5	2.2	2.47	2.8	1.88	1.91	1.96	1.95	1.98	2.05	2	2.1	2.2
	% OMC	14.3	10.7	13	12.8	12.5	9.1	8	7.8	10	9.5	8.5	11	9.9	8.8	11.6	11	9.5	12	13.8	14.6
	% swelling	4.31	0.00	3.12	2.85	2.50	2.16	1.98	1.55	1.55	1.47	1.26	2.20	2.03	1.95	2.60	2.16	2.02	2.25	2.08	1.80
	AASHTO	A-7-6	A-3	A-6	A-6	A-2-4	A-2-4			A-2-4			A-2-4			A-2-4			A-2-4		
	specification																				
	**pH**	7	7.4	7.6	7.8	8.1	12.3	12.4	12.6	12.8	12.9	13.1	7.1	7.2	7.3	7.4	7.5	7.5	6.9	6.7	6.6
		Neutral				Alkaline (basic)													Acidic		

**Table 2:** Specifications of lime and cement kiln dust.

Chemical Composition	Lime	Cement Dust
% Ca(OH)_2_	70–85	—
% SiO_2_	≥2%	11.9
% MgO	≥1%	1.7
% Fe_2_O_3_	≥0.5%	3.4
% Al_2_O_3_	≥0.5%	9.9
% CaCO_3_	≥15%	—
% H_2_O	0.5–1.05	—
% SO_3_	—	1.48
% Na_2_O	—	0.5
% K_2_O	—	0.1
% CaO	—	55.06

**Table 3:** Effects of lime and cement kiln dust on shear strength parameters (C and Φ).

Sample	Cohesion kPa	Friction Angle (Φ)°	Shear Strength τ = C + tan (Φ)
S0	72	21	72.38
S3	86	34	86.67
S6	102	38	102.78
S9	122	44	122.96

## Data Availability

All data presented in this study are available within this article.
